# Molecular epidemiology of human metapneumovirus (hMPV) in Cambodia

**DOI:** 10.1186/1753-6561-5-S1-P71

**Published:** 2011-01-10

**Authors:** Alicia Arnott, Mardy Sek, Sirenda Vong, Philippe Buchy

**Affiliations:** 1 Institut Pasteur in Cambodia, Phnom Penh, Cambodia

## Background

First identified in 2001, human metapneumovirus (hMPV) is a novel pathogen and causative agent of acute respiratory tract infection. Re-infection with hMPV is common, and currently there is no available vaccine against this virus. Two genetic subgroups (A and B) have been identified, both of which can be divided further into at least two distinct sub-lineages. Here we report the results of the genetic variability of hMPV strains circulating within Cambodia.

## Methods

A total of 3858 samples were collected, between 2007-2009, from hospitalized patients presenting with acute lower respiratory illness in the regional hospitals of Takeo and Kampong Cham, under the guidelines of the SISEA (Surveillance and Investigation of Epidemic Situations in Southeast Asia) project. Nasopharyngeal samples were tested for 18 respiratory viruses using multiplex PCR/RT-PCR at the Institut Pasteur in Cambodia. To investigate the genetic heterogeneity of hMPV strains in Cambodia, regions within the highly variable G gene, and highly conserved F gene of 65 strains were amplified. Neighbour-Joining trees were constructed using Mega 4.0 software.

## Results

Most of hMPV cases occurred in 2008 (89%) with a peak in August (32%). Only a very low number of hMPV positive samples were detected in November and December 2007 and in 2009. Partial F gene sequences were obtained from 48 samples, collected from patients between 3 months and 58 years of age. Phylogenetic analysis revealed the co-circulation of B2 (91%) and A2 (9%) genotypes. Genotypes A1 and B1 were not detected. Twenty eight partial G gene sequences were generated. Phylogenetic analysis results were consistent between the F and G genes. We observed more than 10% diversity at the nucleotide level between G gene sequences clustering within the B2 genotype.

## Conclusion

This is the first study to investigate the molecular epidemiology of hMPV in Cambodia, and the first study to report a predominance of circulating genotype B2 strains in Southeast Asia. Our findings contribute to those of recent studies, demonstrating the high variability of the global distribution of hMPV genotypes. This emphasizes the need for ongoing genetic characterization of circulating strains to aid generation of an effective vaccine.

